# Acknowledgment to Reviewers of *Children* in 2020

**DOI:** 10.3390/children8010045

**Published:** 2021-01-14

**Authors:** Children Editorial Office

**Affiliations:** MDPI AG, St. Alban-Anlage 66, 4052 Basel, Switzerland

Peer review is the driving force of journal development, and reviewers are gatekeepers who ensure that *Children* maintains its standards for the high quality of its published papers. Thanks to the cooperation of our reviewers, in 2020, the median time to first decision was 18 days and the median time to publication was 42 days. The editors would like to express their sincere gratitude to the following reviewers for their precious time and dedication, regardless of whether the papers were finally published:
Aadland, EivindLucchese, AlessandraAbalasei, Aurelia BeatriceLuis Ubago, JoseAbasse, SoumethLukowski, Angela F.Abecasis, FranciscoLuna, Ruth AnnAgbangla, NounagnonLund, JonathanAgoston, Anna MonicaLuque Moreno, CarlosAgrawal, PriyankaŁuszczki, EdytaAguaded-Ramírez, EvaLysecki, DaveAguayo, Beatriz BerriosMa, SihuiAhmad, TanveerMaatouk, IsmaelAhmed, Mohamed Lemine Cheikh BrahimMaclellan, Reid A.Akangire, GangaramMacLeod, StuartAlaofe, HalimatouMacMillan, FreyaAlbadri, SondosMahran, AmrAlbright, DanaMair, JacquelineAlinde, KeegstraMakanji, RikeshAllegaert, KarelMalik, TariqAlsharairi, NaserMalin, KatrinaAlsina, MiguelMałysz-Cymborska, IzabelaAlves-Guerreiro, JoseManali, EffrosyniAnanieva, ElitsaMancinelli, ElisaAndersson, Matthew A.Manco, LicínioAngel, Herrera-GuerraMandelbaum, DavidAntoniou, PanosMannion, ArleneAntonogeorgos, GeorgeManolopoulos, AnnaAnzelewicz, Stefan MichałMantziari, AnastasiaArany, EdithMarcão, AnaAritio-Solana, RebecaMarchesi, AlessandraArnbjörnsson, EinarMarlu, RaphaëlArufe Giraldez, VictorMarshall, Teresa A.Aslanidis, TheodorosMartín Valero, RocíoAssari, ShervinMartin, Erin I.Astrid, BertscheMartin, RyanAuten, Richard L.Martin, SarahÁvila-García, ManuelMartina, SandonáAvtanski, DimiterMartinez Selva, DawidAyllón, EsterMartinez-Carrión, José-MiguelAyuso, Juan MielgoMartins, IanBacchetta, RosaMartinuzzi, AndreaBachou, HanifaMascherini, GabrieleBaddock, Sally A.Masetti, RiccardoBader, PeterMason, CarolynneBahmad, HishamMaspero, CinziaBahn, Geon HoMathew, MonaBaía, InêsMathew, RajammaBaile Ayensa, José IgnacioMattsson, JanetBaj, JacekMatziou, VasilikiBajwa, AmandeepMbogori, TeresiaBaldwin, Heather J.McCartan, ClaireBalestrino, RobertaMcCarthy, KevinBang, Kyung-SookMcClafferty, HilaryBarker, AlanMcClain, Kenneth L.Barnes-Josiah, DeboraMcConnell, Patrick I.Bart, OritMcDowell, MichaelBartkowski, JohnMcLafferty, SaraBasset, David R.Mecías-Calvo, MarcosBassi, Carl J.Meddings, DavidBazylak, GrzegorzMei-heng, LinBeauchaine, Theodore P.Mena-Tudela, DesiréeBecker, GenevieveMenon, Renuka T.Behrman, AndreaMentis, Alexios Fotios A.Beinvogl, BeateMerks, PiotrBekteshi, SarandaMerquiol, FanetteBell, LauraMerrick, Jillian S.Bellato, AlessioMester, AlexandruBelteki, GusztavMiçooğulları, Bülent OkanBen-Avie, MichaelMiddleton, MarkBender, Steven D.Migita, OhsukeBerbrayer, DavidMikhael, MichelBergstraesser, EvaMileti, IlariaBerkers, RuudMiller, Jillian VinallBerthon, BronwynMinotti, BrunoBestman, AmyMitchell, Wendy G.Bielinis, ErnestMitra, JoyBierens, JoostMiyagi, HisayukiBignardi, ElioMiyamura, TakakoBinder-Heschl, CorinnaMiyoshi, TakekazuBinford, WarrenMize, MinnieBingham, JennyMocny-Pachońska, KatarzynaBiniwale, Manoj A.Moding, KameronBlack, William RobertMolcho, MichalBlackmore, MarieMolitor, SabineBláha, LadislavMonaro, MerylinBoggero, IanMondanaro, JohnBoles, Jessika C.Mongkuo, Maurice Y.Bolton Moore, CarolynMonsarrat, PaulBonam, Srinivasa ReddyMontanino, MarioBonilla, Diego A.Montemurro, NicolaBooker, CaraMoore, KristinBorsani, OscarMorabito, AntoninoBorzì, FrancescaMoral-García, José EnriqueBothe, DeniseMorand-Beaulieu, SimonBrand, SergeMorasiewicz, PiotrBraun, DavidMorgan, JillBraun, Rudolf K.Morgan, KellyBremer, AndrewMori, MasaakiBrewer, Hannah J.Morris, JoanBrinkman, SallyMoses, JonathanBritt, Rodney D.Motamed, CyrusBronikowski, MichałMoutelíková, RomanaBrottman, GailMuego, MelissaBrunelli, SandroMuiesan, Maria LorenzaBryden, PamMukherjee, ShibabrataBryndal, AleksandraMulcahy Levy, Jean M.Buckler, E. JeanMuñoz, Cynthia E.Bujak-Gizycka, BeataMunoz, Flor M.Busjahn, AndreasMuñoz-López, FranciscoByoung-Hee, LeeMyers, SamuelCadime, IreneNahm, Francis SahngunCai, DeminNair, JayasreeCalcagni, GiulioNajafi, NadiaCallister, Lynn ClarkNakata, YoshioCameron, NoelNam, Ok HyungCampus, GuglielmoNamba, FumihikoCanciani, ElenaNandalike, KiranCapelli, GiovanniNandi, DeipanjanCaridade, Sónia Maria MartinsNarkewicz, Michael R.Carli, MarcoNatarajan, Sathish KumarCarmona-Mora, PaulinaNath, AudreyCarpinelli, LunaNelson, Sarah M.Carrouel, FlorenceNemeș, Roxana MariaCarter, BernieNeves, RuiCarter, Brian SNewman, ChristopherCasas, Rosa M.Nguyen, Hoang H.Castillo, SilviaNguyen, ThinhCastillo-Rodríguez, AlfonsoNicholson, MaribethCastro-Alonso, CristinaNieminen, PenttiCatto-Smith, AnthonyNiermeyer, SusanCerník, DavidNjoku, AnuliChalkley, AnnaNoble, FionaChallagundla, KishoreNordström, ThomasChampagne, CatherineNota, AlessandroChan, Chin-KanNotas, GeorgeChan, Ernest G.Nouri, AriaChan, Kak-ChenNoviello, CarmineChandrasekharan, Praveen K.Nowak, Paweł FryderykChawner, SamuelNsabimana, EpaphroditeChen, ChaoNucera, RiccardoChen, Chiao-NanNwabudike, Lawrence ChukwudiChen, Chi-NienOberle, MeganChen, Gwo-ShingOei, Ju LeeChen, Kong Y.Ohliger, ShelleyChen, YangO’Leary, FentonChen, Yueh-ChihOledzka, MagdalenaCheng, HaiziOliva, AntonioChiappedi, MatteoOliván-Gonzalvo, G.Chien, Yin-HsiuOliva-Pascual-Vaca, JesúsChillón Garzon, PalmaOppici, LucaChing, ChristinaOsooli, MehdiChisari, EmanueleOtsuka, MitsuoChiu, Hsiu-ChingOuellet, MarilouChmelik, FrantisekOwens, MattChoisne, JulieOwili, Patrick OpiyoChou, Pang-YunOzanne-Smith, JoanChristian, DanielleOzbič, MartinaChu, Yen-JuPaap, Kenneth R.Chuang, Hai-HuaPacini, MatteoChutiyami, MuhammadPaczkowska-Abdulsalam, MagdalenaClark, AndrewPadmanabhan, PradeepClemente, Maria GraziaPalakurthi, BhavanaCollins, Andrew B.Pan, YijunCollins, ChristopherPanaitescu, AncaColombo-Dougovito, Andrew M.Pangrazzi, LucaCompagnon, RoxanePapadopoulou, MariannaConneely, Shannon E.Papakonstantinou, EvgeniaConstantinescu, AlexandruPapamitsou, TheodoraCorbett, Blythe A.Paprocka, JustynaCorrell, LynniePark, Eun-YoungCounil, Francois-PierrePark, JusikCoussens, ScottPark, Sook-HyunCozzani, EmanueleParker, Alasdair Pj.Crane, Jeff R.Parodi, AuroraCriss, ShaniecePasman, Wilrike J.Critser, Paul J.Paterson, Rebecca S.Cross, ChristinaPatiño, María José MartínezCrowley, JamesPatro-Małysza, JolantaCrume, Peter K.Paweł, TomaszewskiCuenca, AlexPearl, PhillipCuesta Gómez, AliciaPearn, JohnCurtis, JacquelinePeila, ChiaraCurto, AdriánPeng, MeiCutrona, CarolynPerdikaris, PantelisCzepczor-Bernat, KamilaPereira, ArmandaD’Amario, MaurizioPeressotti, FrancescaD’Souza, GenevievePérez-Bermejo, MarcelinoDachsangvorn, OnaPérez-Fuentes, María Del CarmenDalal, Priti G.Perin, CeciliaDalimonte-Merckling, DaniellePerry, Sarah E.Dall’Oglio, LuigiPesavento, RussellDamtie, DebasuPetca, RǎzvanDanysh, Heather E.Peter, MetcalfeDas, AbhirupPeterson, CatherineDavies, W. HobartPetousi, VasilikiDawes, ColinPeyer, KarissaDawes, PatrickPhilip, Roy K.Day, KaitlinPiątkowska, MonikaDe Almeida Honório, Samuel AlexandrePickett, Andrew C.De Gennaro, LuigiPicoraro, Joseph A.De La Fuente-Solana, Emilia I.Piersigilli, FiammettaDe La Vega De Carranza, RocíoPinheiro, FabioDe La Vega, RocíoPinheiro, JoaquimDe Leeuw, PeterPitti, Julio AlvarezDe Müllenheim, Pierre-YvesPlevinsky, JillDeacy, AmandaPogorelić, ZenonDeCocker, KatrienPokorska-Niewiada, KamilaDeeb, WissamPop, Tudor LucianDel Barba, PaoloPopiołek-Kalisz, JoannaDel Pozo De La Calle, SusanaPoulimeneas, DimitrisDelaney, CassidyPrasad, Amit A.Delaroche, Amy M.Preez, Surita DuDenis, FredericPróchniak, PiotrDeutsch, NinaProcino, GiuseppeDevlin, Lori A.Proctor, Mark R.Di Lorenzo, MarianaProsperi, MargheritaDi Nardo, MatteoPrzybyła-Basista, HannaDi, SharonPulido, OlgaDiogo, EliseteQadri, GhalibDixit, Anjali A.Qvist, NielsDixon, AndrewRacil, GhaziDiykh, MohammedRadnor, John M.Dodero, VeronicaRadoja, IvanDohme, Lea-CathrinRamamurthi, RamDoi, TakehikoRamirez-Graniz, Irwin AndrésDoll, DietrichRamiro-Cortijo, DavidDonohue, Lee TRanabhat, ChhabiDorota, GaskinsRanjith, KamityDowell, Richard C.Rapkin, LouisDriessen, GeertRascher, WolfgangDudeney, JoanneRaveendran, SanjeethaDudeney, Joanne E.Rawat, MunmunDun, Matthew D.Rehman, LaureneDuncanson, KerithRehman, MichaelDussel, VeronicaReis Gaya, AneliseDutra, Samia V.Reiss, Allan L.Dynia, JaclynRenate, FuikoEaton, SimonResiere, DaborEdens, CuoghiReula, AnaElce, AusiliaRichards, NoniEliades, TheodoreRichardson, Patricia A.Elias, AmanuelRiedel, ThomasElisabetta, Cretella LombardoRiley, NicholasElliott III, WilliamRima, SamyEngelhardt, Thomas E.Roberts, Michael W.Esposito, CiroRobertson, ScottEsteban-Gorgojo, IgnacioRobinson, SoniaEvers, Patrick D.Rodrigues, CarinaFalsaperla, RaffaeleRodríguez-Almagro, DanielFang, DiRolle, UdoFasoli, Allison DiBiancaRomano, AlbertoFavilli, SilviaRomeo, DeborahFeig, EmilyRomeo, DomenicoFeinstein, JamesRoscoe, ClareFeldkamp, MarciaRosi, EleonoraFeldman, Jacob I.Ross, JenniferFelknor, Sarah A.Ross, JulieFernández Carnero, SamuelRothe, ThomasFernandez, MercedesRothschild, BruceFerrandiz, CarmenRozensztrauch, AnnaFerrara, FelicettoRozerta, SokouFerrara, PietroRudolph, HenrietteFerrer-Sargues, Francisco JoséRustico, LorenzoFiedorowicz, EwaRusy, Lynn M.Fink, James B.Ryu, Ji-wonFinley, AllenSabbagh, CharlesFiori, CristianSacchi, ChiaraFlores-Fraile, JavierSakamoto, RyuichiFloríndez, Lucía I.Sakhnini, VictoriaFlynn, EmileeSalamon, Katherine S.Fonnes, SivSalö, MartinForget, PatriceSaltaji, HumamForsum, Elisabet A.Salvador-Coloma, PabloFoulkes, JonathonSamadi, Sayyed AliFrader, Joel ESamppa, RyhanenFreitas, RaquelSánchez-Miguel, Pedro AntonioFriedman, CliveSánchez-Sánchez, EduardoFusar-Poli, LauraSánchez-Zafra, MaríaGagnon, Joseph CalvinSanchis-Soler, GemaGalan-Lopez, PabloSanfi, IlanGaleotti, FabioSani, AnaGarcía-Galbis, Manuel ReigSankararaman, SenthilkumarGarcía-Garnica, MarinaSankarasubramanian, VishwanathGarcía-Muñoz Rodrigo, FerminSansone, FrancescoGarcía-Soidán, Jose L.Santos, Maria PaulaGaribotto, GiacomoSantos, SofiaGarriboli, MassimoSantos-Lozano, AlejandroGatti, FloraSar, Bibhuti K.Gavini, MadhaviSaraiva, LindaGawlowski, PawelSathish, ThirunavukkarasuGaze, MarkSatoh, TarohGelzo, MonicaSavant, AdrienneGeoffray, Marie MaudeScala, MelissaGeorge, KoumantakisScharoun Benson, SaraGepner, YftachSchechter, NeilGerson, TrevorSchechtman, SamuelGiacani, LorenzoScherzer, Daniel J.Gibson, PhilippaSchlegel, Peter N.Gien, JasonSchmidt, AndrewGierczyk, MarcinSchmidt, Sabrina KroghGigante, AntoniettaSchmitt-Wilson, SarahGiles, AudreySchroeder, KristaGiontella, AliceScrimshire, AshleyGlenister, KristenScurati, RaffaeleGodoy-Izquierdo, DéboraSederstrom, NnekaGoggin, Maureen DalySerbic, DanijelaGomes, Thayse NatachaSerra-Paya, NoemiGómez-Baya, DiegoSeverino, PaoloGómez-de-Terreros-Guardiola, MontserratSfeatcu, Ionela RuxandraGómez-Tafalla, AnaShah, RugminiGonzález Azpeitia, GloriaShahmoradi, MahdiGonzález, DanielShamshirsaz, Alireza A.Gonzalez-Alonso, FernandoShankland, RebeccaGonzalez-Jurado, Jose AntonioSharma, Adhikarimayum LakhikumarGoodwin, MichaelaSharma, AtulGordish-Dressman, HeatherSharma, SapnaGori, DavideSharma, VinayGorodzinsky, AyalaSharmin, SoniaGosav, Evelina MariaSharon, DeniseGoudas, MariosSherrington, Anna MariaGounaris, Antonios K.Shiao, Chih-ChungGraham, TiffanyShim, Jung OkGreenhalgh, DavidShin, DongheeGribble, KarleenShindova, MariaGrillo, AndreaShinnar, ShlomoGroba, BetaniaShinsugi, ChisaGu, XiangliShkodra, MorenaGuevara, JenniferShulman, Stanford TGuinsburg, RuthSiafis, SpyridonGupta, MedhaviSiegel, Robert M.Gustafsson, Nina-KatriSilva, Maria-Raquel G.Gutíerrez Hervas, Ana IsabelSilvia, PaulGuynes, KristenSimioni, CarolinaGwynette, McLeodSimone, PisanoHaapa, ToniSingh, Ajay PratapHakeem, Faisal F.Singh, RajbirHalley, MeghanSinnott, Catherine J.Halski, TomaszSisk, CaraHan, SungilSiziba, LindaHanke, WojciekSkinner, ChristopherHanna, AmgadSlee, PhilllipHaro, GonzaloSleet, DavidHarrington, Deirdre M.Slezak, RyszardHart, LaraSlusher, Tina M.Harton, AnnaSmith Roley, SusanneHausser, IngridSmith, AllisonHeaton, Steven M.Snyder, JulieHegyi, Thomas HegyiSobey, JennaHeinen, MirjamSofo, SeiduHerbert, AnthonySommer, GritHerbuela, Von Ralph Dane M.Song, In GyuHernandez Fernandez, AntonioSourial, Maryanne Y.Hernandez, ErikaSpivakovsky, SilviaHernández-Alonso, PabloSprague, WendyHerrero, OscarSreedher, GayathriHerzog, SarahSribnick, Eric A.Hida, AzumiStaderini, EdoardoHidalgo Lopezosa, PedroStalsberg, RagnaHillman, HeidiStaunstrup, Nicklas HeineHirsch, ChristianStavinoha, Peter L.Ho, Pei-ShanStefani, LauraHoellwarth, JasonStewart, ThomasHojan, KatarzynaStiver, CoreyHolt, KellyStokes, LynissaHomma, TetsuyaStone, AmandaHong, Bo YoungStraburzynski, MarcinHopp, RussellStrunecka, AnnaHorhat, DeliaSu, ErikHowerton-Fox, AmandaSun, NaiyiHsu, Li-ChiSurendran, SushithaHuang, CharlesSvoboda, LaurieHughes, Christopher V.Sweeney, BrookeHughes, Jean R.Tagg, AndrewHügle, BorisTaked, TakayukiHugrass, LailaTamir, Sharon OvnatHuisman, SylviaTammelin, TuijaHulteen, RyanTang, JingjingHurtado, Ghaffar AliTanoubi, IssamHusain, RalfTarraga-Minguez, RaulHyun, TaisunTaylor, Danielle H.ILAND, HARRYTaylor, GreigIlyas, MohammedTe Nijenhuis, JanInes, Van KeerTeilmann, Grete KatrineIng, Frank FTeng, Ru-JengInnerd, AlisonTennant, MarcIorio, RaffaeleTeran-Garcia, MargaritaIshii, YumikoThavamani, AravindIskusnykh, Igor Y.Theorell-Haglöw, JennyIzzo, RiccardoThielen, BethJabłonowski, ZbigniewThomas, Pauline A.Jacob, JennaThompson, DeborahJácome, CristinaTichko, ParkerJacques, AngelaTilga, HenriJaehne, Anja KathrinTimmers, IngeJälevik, BirgittaTimpani, CaraJancelewicz, TimTing, Chien-KunJanowski, MiroslawTo, YasuoJanssens, ThomasToczylowski, KacperJanuszkiewicz-Lewandowska, DanutaToida, ChiakiJasien, JoanTolaymat, AbdullahJasiewicz, BarbaraTomczak, AndrzejJayaprakash, PriyamvadaTomczyk, ŁukaszJenkins, Todd M.Tong, Yat-ChingJillella, DineshTóth, ÁkosJones, Paul C.Toyoda, HidemiJones, RachelTraczyk, IwonaJoronen, KatjaTrahair, TobyJose, PerezTrajanoska, KaterinaJuárez-Ruiz De Mier, RocíoTrakada, GeorgiaJungquist, Carla R.Tran, Susan T.Jurjans, KristapsTrip, SimonaKaczyńska, KatarzynaTripathi, Jitendra KumarKaditis, AthanasiosTriunfo, StefaniaKaiser, PamelaTruong, UyenKaissi, Ali AlTryggestad, JeanieKaminska, MargaretTsimpli, Ianthi MariaKampouras, AsteriosTsytsarev, VassiliyKan, Pui FongTurkmen, MutluKanakri, ShireenUmpaichitra, VatcharapanKane, KyraUrv, Tiina K.Kang, Augustine W.Valitutti, FrancescoKao, GraceVan Arendonk, Kyle J.Karasik, Lana B.Van Baar, AnneloesKarisola, PiiaVan Bergen, ChristiaanKasper-Jędrzejewska, MartynaVan Der Helm, PeerKatherine, ProwseVan Der Mark, EliseKatz, PhillipVan Dijk, MoniqueKatz, TerryVan Kann, DaveKawamura, TomoyukiVan Kann, Dave (D.H.H.)Keil, Margaret F.Van Tilburg, Miranda A.L.Keisaku, SatoVanderloo, Leigh M.Kendall-Taylor, NathanielVatsayan, AnantKent, JacquelineVeazey, Leah WilliamsKent, JenniferVemuri, RavichandraKesavan, KalpashriVerrastro, ValeriaKevin, AckermansVesoulis, ZacharyKhalil, NadimVidaurri, LytorreKhanday, Mudasir A.Vieira, MargaridaKhubchandani, JagdishVillani, CiroKim, HyungjooVirella, DanielKim, JiyongVittori, AlessandroKim, Sang-DolVolochayev, RitaKing, Christopher D.Von Döbeln, UlrikaKnisel, ElkeVon Heideken, JohanKobel, SusanneVoss, Megan E.Koechlin, HelenVuille-dit-Bille, Raphael NicolasKolodziejski, Pawel AntoniWager, JuliaKorcz, AgataWagner, JessycaKorinthenberg, RudolfWahl-Alexander, ZacharyKosanovic, DjuroWalsh, ShanaKotzalidis, Giorgio D.Walsh, Stephanie M.Koutelidakis, Antonios E.Walters, Camila B.Kovacic, Katja KWang, AlbertaKozakiewicz, MarcinWang, CynthiaKozioł-Kozakowska, AgnieszkaWang, JianxiongKozlik-Feldmann, Rainer GerhardWang, LeiKrajnak, Kristine M.Wang, Tang-ChuanKristen (Kris), DovganWard, Kenneth D.Krüger, BjörnWarner, John O.Krupp, Lauren B.Warschburger, PetraKumar, Kishore RajWasilewska, ElizaKumar, Maya M.Wasniewska, MalgorzataKumar, SuneelWatanabe, JunKuo, Hsien-WenWatanabe, KenichiroKuppusamy, ManiselvanWatson, MichaelKurata, MieWawrzyniak, AgataKurosaka, HiroshiWeingarten, KevinKushner, Brian H.Weledji, Elroy PatrickKyvelidou, AnastasiaWeres, AnetaL. Wood, MeganWeyand, Angela C.La Mantia, IgnazioWhitaker, Amanda T.Lăcătușu, Cristina-MihaelaWhite, HankLack, SharonWhite-Lewis, SharonLademann, HanneWiart, LesleyLamba, VineetWickström, RonnyLander, NatalieWijesinghe, Ruchire ErangaLandis, Carolyn E.Williams, AlisonLandt, EskildWilliams, Cydni N.Lane, GinnyWilliams, MarcLane, Shelly J.Williams, SaraLange, JoannaWilliams, Sara E.Lanks, Charles W.Williamson, PaulLara Sánchez, Amador JesúsWilson, Jenny L.Laudanska-Krzeminska, IdaWithrow, NicoleLawson, Nicholas D.Włodarek, DariuszLebon, SébastienWojciechowski, WallyLee, Hui JaiWong, WilliamLee, Jason JiunshiouWood, Thomas R.Lee, JoonyoungWoods-Townsend, KathrynLee, Jung EunWu, Chiao-EnLee, JustinWu, Zhi-FuLee, YoonJungXie, ZhiqunLefere, SanderXu, FurongLemanek, KathleenYamauchi, Melissa S.W.León-Quismondo, JairoYanai, KazuhikoLera, María JoséYang, Hui-TingLerma, ClaudiaYang, JiminLeva, ErnestoYang, Shin-seungLevine, ShelYarger, HeatherLi, Chia-JungYen, Cheng-ChiehLi, Dian-JengYen, Cheng-FangLi, ShuaiYokomichi, HiroshiLi, TengfeiYousefzadeh, SepidehLichenstein, RichardYu, Hong-RenLin, Hui-ChuZadra, CinziaLin, Yuan-ChiZagalaz Sánchez, María LuisaLin, Yuh-JyhZaghloul, NahlaLin, Yung-ChiehZakrocka, IzabelaLlopis-González, AgustínZaman, WidaadLober, AngelaZambrano, Rodrigo ElíasLocher, CosimaZanotto, LuciaLombardi, MarcoZarantonello, PaolaLombardi, TommasoZernikow, BorisLongobardi, ClaudioZhang, YinfengLonhart, Julia A.Zhao, BaoyinLopez, AntonellaZheng, Quan TohLorås, HåvardZolnikov, Tara RavaLourenço, OlgaZotti, Francesca

